# High-grade HER2-positive mucoepidermoid carcinoma of the breast: a case report and review of the literature

**DOI:** 10.1186/s13256-023-04233-0

**Published:** 2023-12-08

**Authors:** Mario Della Mura, Céline Clement, Maria P. Foschini, Sara Vander Borght, Lise Waumans, Peter Van Eyken, Esther Hauben, Machteld Keupers, Caroline Weltens, Ann Smeets, Ines Nevelsteen, Giuseppe Floris

**Affiliations:** 1 Department of Imaging and Pathology, Laboratory of Translational Cell & Tissue Research, KU Leuven—University of Leuven, 3000 Louvain, Belgium; 2 Department of Pathology, KU Leuven—University of Leuven, University Hospitals Leuven, Herestraat 49, Campus Gasthuisberg, 3000 Louvain, Belgium; 3 School of Medicine and Surgery, Magna Græcia University of Catanzaro, Viale Europa, Germaneto University Campus, 88100 Catanzaro, Italy; 4 Department of Oncology, KU Leuven—University of Leuven, 3000 Louvain, Belgium; 5 Department of Surgical Oncology, KU Leuven—University of Leuven, University Hospitals Leuven, 3000 Louvain, Belgium; 6 Department of Biomedical and Neuromotor Sciences, Unit of Anatomic Pathology, University of Bologna, Bellaria Hospital, 40139 Bologna, Italy; 7 Department of Pathology, Regional Hospital East Limburg (ZOL), 3600 Genk, Belgium; 8 Department of Radiology, KU Leuven—University of Leuven, University Hospitals Leuven, 3000 Louvain, Belgium; 9 Department of Radiotherapy Oncology, KU Leuven—University of Leuven, University Hospitals Leuven, 3000 Louvain, Belgium

**Keywords:** Triple-negative breast cancer, Salivary gland-like tumors of the breast, Mucoepidermoid carcinoma of the breast, HER2, Case report

## Abstract

**Background:**

Mucoepidermoid carcinoma of the breast is a rare special type of salivary gland-like tumor of the breast, usually displaying triple-negative phenotype. To date, only 64 cases have been reported in the English literature. Herein, we report the first case of mucoepidermoid carcinoma of the breast with human epidermal growth factor receptor 2 gene amplification.

**Case presentation:**

A 58-year-old Caucasian woman treated with breast-conserving surgery, radiotherapy, and chemotherapy for an invasive breast carcinoma of no special type, relapsed 20 years later in the ipsilateral left breast. Histological examination of the core needle biopsy of the relapse deferred to the surgical specimen for the definitive diagnosis, because of the broad differential diagnosis. On the resected specimen we observed the presence of a poorly differentiated carcinoma with mucoepidermoid carcinoma of the breast typical features consisting of epidermoid, intermediate and mucinous cells lacking true keratinization, in keeping with the latest World Health Organization diagnostic criteria. The mucoepidermoid carcinoma of the breast was weakly estrogen receptor and androgen receptor positive and progesterone receptor negative, but exceptionally showed human epidermal growth factor receptor 2 gene amplification. Mastermind-like transcriptional coactivator 2 gene translocations were not detected by fluorescent in situ hybridization. The patient received adjuvant chemotherapy with anti-human epidermal growth factor receptor 2 therapy but no endocrine therapy. After 61 months of follow-up, no signs of local or distant recurrence were observed.

**Conclusions:**

Mucoepidermoid carcinoma of the breast is a very rare entity. Despite being most frequently triple negative, the standard evaluation of receptor status is mandatory, as well as strict application of World Health Organization diagnostic criteria for correct patient management.

## Background

Mucoepidermoid carcinoma of the breast (MEC-b) is a rare special type of breast carcinoma (BC) accounting for < 1% of all breast malignancies, and belonging to the salivary gland-like tumors. Despite being mostly classified as triple negative breast carcinoma (TNBC) it is usually considered a tumor with low-malignant potential and good prognosis [1].

According to the project Surveillance of Rare Cancers in Europe (RARECARE), rare tumors are defined as those with an incidence of < 6/100,000 per year. In 2011 the estimated cumulative incidence of all salivary gland-like tumors of the breast was 0.05/100,000 per year, with a prevalence of about 2400 new diagnoses per year in the whole of Europe [2]. A similar incidence is reported also in the USA, rendering MEC-b an exceedingly rare type of BC [3].

MEC-b is composed by a mixture of mucinous, epidermoid, and intermediate neoplastic cells arranged in solid and cystic structures. Their presence is mandatory for the diagnosis as well as the lack of true keratinization [4]. Grading of MEC-b is done either by using breast cancer criteria (in other words, Nottingham Histologic Score System) or salivary gland cancer criteria (in other words, the Armed Forces Institute of Pathology grading system) [4]. Immunohistochemistry (IHC) is useful and assists with morphology in confirming the diagnosis.

Mastermind-like transcriptional coactivator 2 (*MAML2*) gene translocations have been recently described in some cases, a feature shared with MEC of the salivary glands (MEC-sg) [5–8].

Herein we present a case of recurrent BC showing typical MEC morphology and demonstrating human epidermal growth factor receptor 2 (*HER2*) gene amplification. We also provide a review of the current literature in the view of current World Health Organization (WHO) essential criteria for diagnosis [4]. Given the reported worse prognosis of rare cancers compared with the prognosis of more common cancers [2], we aimed to improve knowledge, and provide clinical guidance for the diagnosis and treatment of such rare cases.

## Case presentation

A 58-year-old Caucasian woman presented to our hospital with a self-palpated mass in the left breast.

The patient was in follow-up since 1996 for a previous BC located in the upper outer quadrant of the same breast: a grade 3 invasive breast carcinoma of no special type (IBC-NST; pT1cN0M0), hormone receptor positive (Allred score: ER 6/8 and PR 7/8) and treated by lumpectomy with axillary lymph node dissection and adjuvant chemotherapy (a-CT) (six cycles of cyclophosphamide, methotrexate, and 5-fluorouracil) followed by radiotherapy (breast 50 Gy + 16 Gy boost) without endocrine therapy. Beside the presence of breast cancer in a second degree female relative (father side) older than 55 years, no further breast- or ovary-related tumors were retained in her family. Her mother died from bladder cancer.

**Fig. 1:**
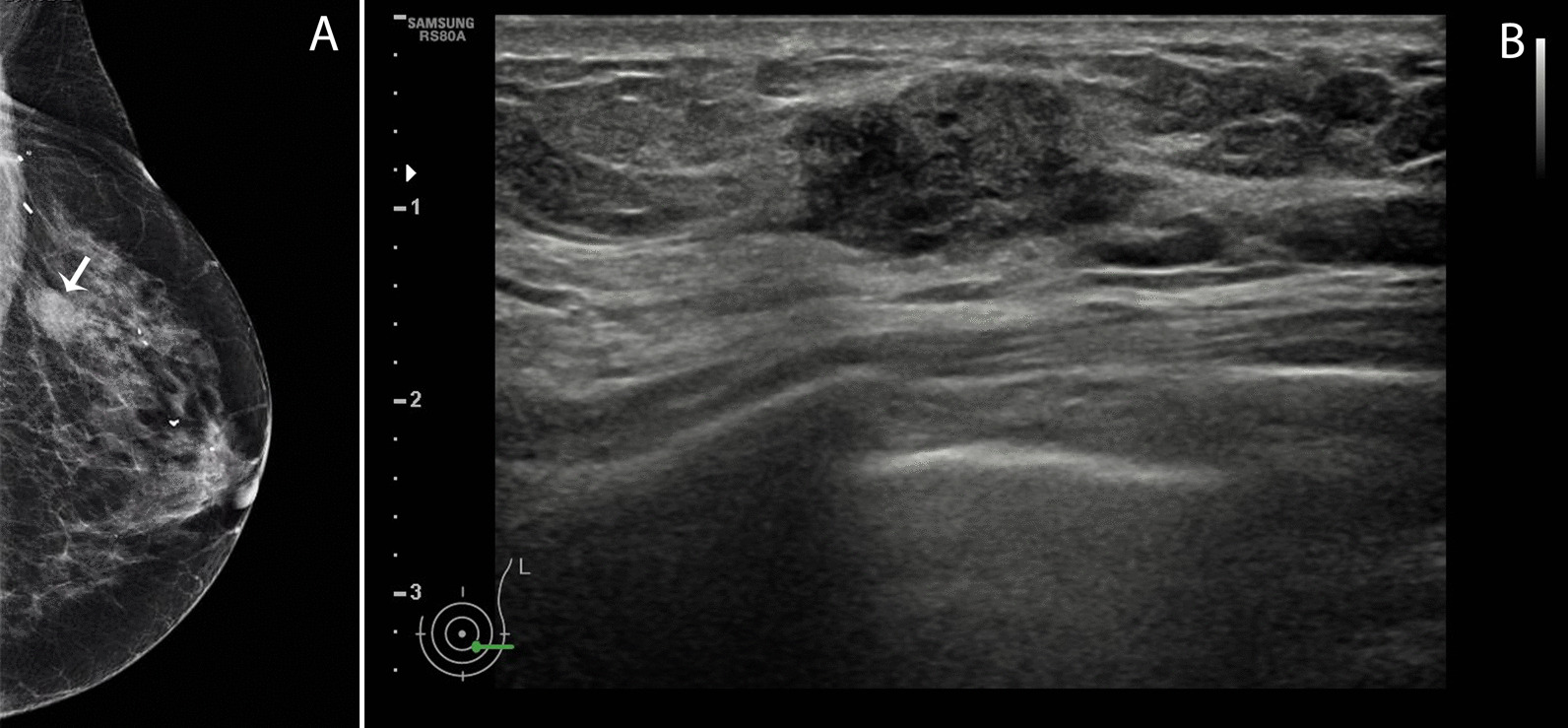
RX and ultrasound imaging of the left breast. **A** On mammography, the cranio-caudal (CC) prospect shows the presence of a deeply located nodular shaped dense mass at 3 o’clock with irregular borders and highly suspicious of malignancy (white arrow). Sequelae of the previous surgery are visible as well. **B** On ultrasound the lesion was hypoechoic showing parallel orientation, irregular contours and heterogeneous composition

Clinical examination confirmed the presence of an irregular nodule localized at 3 o’clock, which by palpation measured 30 mm × 25 mm in size, free from the skin and the pectoral muscle, without lymphadenopathy. Mammography showed an irregular dense mass of 18 mm × 14 mm highly suspicious for malignancy, and ultrasounds showed a hypoechoic mass with parallel orientation, irregular contours, and heterogeneous composition (Fig. 1). On core needle biopsy a high-grade invasive BC with eosinophilic cells suspicious for squamous/epidermoid or apocrine differentiation without mucinous component was described, deferring definitive diagnosis to the surgical specimen (not shown). Standard staging with chest X-ray, abdominal ultrasound, and skeletal scintigraphy excluded the presence of distant metastasis. The patient underwent to a simple left mastectomy for a rcT1NxM0 BC.

**Fig. 2:**
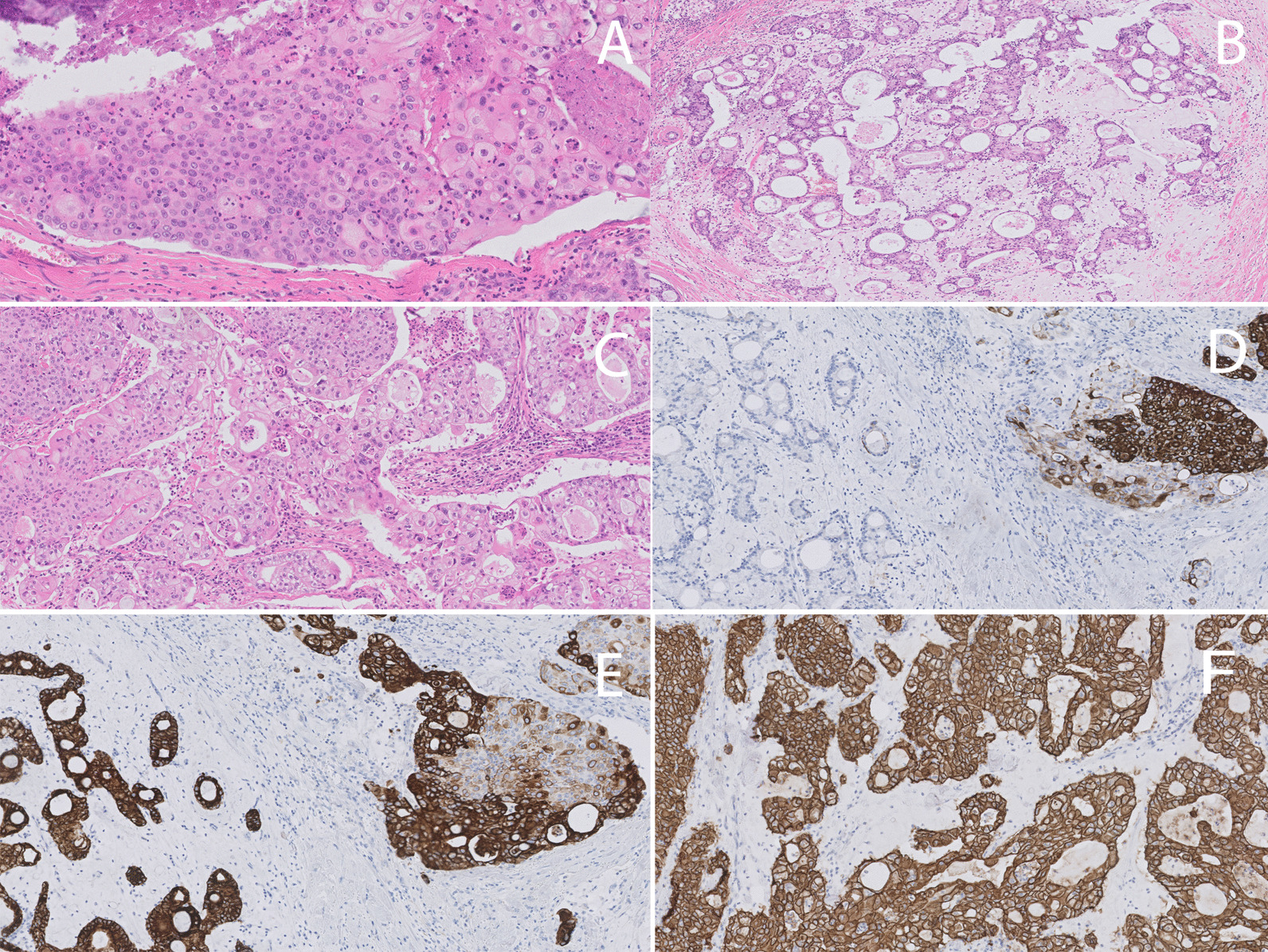
microscopic features of MEC-b [hematoxylin and eosin (H&E) and immunohistochemistry]. **A** The tumor cells were mostly arranged in solid nests admixed with necrotic areas (upper left and right corner). The tumor cell population was characterized by a mixture of large epidermoid cells and relatively small intermediate cells in absence of mature keratinization. **B** Microcystic and cribriform structures lined by tumor cells with mucinous differentiation, floating in large pools of extracellular mucine. **C** At high power magnification the heterogeneous tumor cell composition was clearly visible. Next to large epidermoid cells and intermediate cells, we noticed also the presence of scattered cells with clear cytoplasm and mucinous differentiation. The adjacent stroma showed moderate mixed inflammatory infiltrate characterized by high number of neutrophils. The formation of several micro-abscesses was also apparent. The typical zoning pattern described in MEC was clearly visible by sequential staining with CK 5.6 (**D**) and CK7 (**E**). The two microphotographies show a mirror picture with large epidermoid cells positive for CK5.6 but negative for CK7, and conversely the mucinous component positive for CK7 but negative for CK 5.6. **F** The HER2 immunostaining surprisingly showed strong and diffuse membranous staining in all tumor cells. Fluorescence *in situ* hybridization (FISH) analysis confirmed the amplification of the *HER2* gene

Gross inspection revealed a sharply demarcated nodular and white tumor of 20 mm diameter. Microscopically, a dominant non-capsulated nodule associated with rare peripherally located lymphoid structures was observed. The tumor cells were mostly arranged in solid nests admixed with necrotic areas. A composite population including large highly pleomorphic epidermoid cells and relatively small intermediate cells with indefinite cell borders and oval-shaped nuclei in absence of mature keratinization was observed (Fig. 2A). Additionally, the presence of cribriform and microcystic structures embedded in large extracellular mucin pools, associated with columnar mucin producing epithelial cells, was noticed as well (Fig. 2B). Frequent micro-abscesses were present (Fig. 2C). We counted up to seven mitoses per mm^2^. Finally, a component of poorly differentiated ductal carcinoma *in situ* (DCIS) with MEC features was also observed.

By IHC the composite mixture of tumor cells was confirmed by a combination of high and low molecular weight cytokeratins (Fig. 2D, E). Areas with epidermoid differentiation showed p63 and GATA3 staining; BRST-2 was negative.

Nuclear weak AR and ER expression was observed in < 10% of the tumor cells in the mucinous component. PR was negative. HER2 showed a score of 3+  (Fig. 2F). Flurescence *in situ* hybridization (FISH) analysis confirmed *HER2* gene amplification and showed absence of *MAML2* rearrangements. The DCIS component was HER2 positive but lacked hormone receptor expression.

The revision of the IBC-NST of 1996 confirmed the absence of MEC features.

The final diagnosis of grade 3 breast MEC was proposed (rpT1Nx).

The adjuvant therapy consisted of paclitaxel (12 cycles, weekly) and trastuzumab (18 cycles, every 3 weeks). Aromatase inhibitors were not administered because of the low ER and potential unfavorable side-effect/benefit ratio. Germ-line genetic screening excluded presence of predisposing mutations for hereditary breast–ovarian cancer syndrome.

After 61 months of follow-up the patient is alive, without any sign of recurrence.

### Methods

The patient provided her informed consent and clinical history and imaging were retrieved from her medical files.

IHC was performed using the following antibodies: ER (Dako, clone EP1, ready to use), PR (Dako, clone PgR1294, ready to use), AR (Dako, clone AR441, dilution 1:100), HER2 (Dako, polyclonal rabbit anti-human c-erB-2 oncoprotein, dilution 1:1000), cytokeratin 5/6 (CK5.6) (Dako, clone D5/16 B4, ready to use), cytokeratin 7 (CK7) (Dako, clone OV-T2 12/30, ready to use), transformation-related protein 63 (p63) (Dako, clone DAK-p63, ready to use), GATA binding protein 3 (GATA3) (Biomedical Care, clone L50-823, ready to use), and gross cystic disease fluid protein-15 (BRST2) (Dako, clone D6, dilution 1:300). The Dako EnVision FLEX Target Retrieval Solution High pH (50×) (Dako Omnis) was used for the antigen retrieval of all antibodies, but for BRST2 EnVision FLEX Target Retrieval Solution Low pH (50×) (Dako Omnis) was used.

FISH for *HER2* [PathVysion HER-2 DNA Probe Kit (PathVysion Kit)] and *MAML2* rearrangements [SPEC MAML2 Dual Color Break Apart Probe (Zytovision)/Histology FISH Accessory kit (Dako)] was performed, following vendors’ specifications.

## Discussion

MEC-b is a rare subtype of TNBC that has morphomolecular features in common with MEC-sg counterpart. Breast and salivary glands are both exocrine glands derived from the embryonal ectoderm, which also explains the shared morphology with MECs from other organs. Herein we present a case of a recurrent BC with typical histopathological MEC-b features, but showing *HER2* amplification.

Only 64 cases of MEC-b have been reported in English literature so far. MEC-b has been described exclusively in females aged from 29 to 86 years (average 59 years) (Table 1). Despite the predominant TNBC phenotype, low grade MEC-b are associated with good prognosis. Interestingly, BC-specific mortality and metastasis seems to occur only in high grade MEC-b, while mortality and metastasis in low- and intermediate-grade MEC-b are absent, even without a-CT [5]. These observations render the role of a-CT questionable in low-grade MEC-b. For this reason, a recent consensus statement endorses the use of tumor grading to inform clinicians about the need of a-CT in MEC-b [9]. Our case showed typical high-grade MEC-b features, using both grading systems for breast and salivary glands [4], supporting the use of a-CT.

**Table 1:** Literature overview of breast MECs

^No^	^Authors^	^Year of publication^	^Age^	^Tumor dimension (cm)^	^Grading^	^Lymph node metastasis^	^Distant Metastasis^	^Type of surgery^	^Adjuvant therapy^	^Follow-up (months)^	^ER−PR status^	^HER2 status^	^Molecular analysis (*MAML2*translocation)^
1	Patchefsky *et al.* [10]	1979	70	5	LG	NA	No	Q	NA	10 alive	NA	NA	NA
2			66	1.3	LG	No	No	RM	NA	94 DOR	NA	NA	NA
3	Kovi *et al*. [11]	1981	46	11	HG	Yes	NA	MRM	NA	NA	NA	NA	NA
4	Fisher *et al*. [12]	1983	60	4	LG	NA	No	SM	NA	48 alive	NA	NA	NA
5			49	3.7	LG	No	No	RM	NA	108 alive	NA	NA	NA
6			57	2.5	LG	No	No	MRM	NA	120 alive	NA	NA	NA
7			71	2	LG	No	No	MRM	NA	48 alive	NA	NA	NA
8			65	2	LG	NA	No	L	NA	60 alive	NA	NA	NA
9	Ratanarapee *et al*. [13]	1983	27	NA	HG	Yes	Yes	NA	NA	14 DOD	NA	NA	NA
10^*b*^	*Leong and Williams* [14]	1985	57	3.5	HC	No	Yes	SM	No	7 DOD	NA	NA	NA
11	Hastrup and Sehested [15]	1985	59	1	HG	No	Yes	RM	RT+CT+HT	25 DOD	−−	NA	NA
12b	*Hanna and Kahn* [16]	1985	31	NA	NA	Yes	No	MRM	CT	14 alive	+−	NA	NA
13^b^			51	2	NA	No	No	MRM	No	8 alive	+−	NA	NA
14^b^	*Pettinato* *et al.* [17]	1989	72	7	HG	Yes	Yes	MRM	CT	10 DOD	NA	NA	NA
15^b^	*Luchtrath and Moll* [18]	1989	60	5	HG	Yes	Yes	RM	NA	30 DOD	NA	NA	NA
16	Chang *et al*. [19]	1998	54	4.5	HG	No	No	MRM	CT	48 alive	NA	NA	NA
17	Markopoulos *et al.* [20]	1998	40	2	HG	No	No	L+ALND	NA	60 alive	NA	NA	NA
18	Berry *et al*. [21]	1998	51	3.5	HG	No	no	MRM	NA	NA	NA	NA	NA
19	Tjalma *et al*. [22]	2002	58	3.5	HG on LG	Yes	Yes	RM	NA	156 alive	NA	NA	NA
20	Terzi *et al*. [23]	2004	79	8	HG	Yes	No	MRM	NA	NA	NA	NA	NA
21	Di Tommaso *et al*. [24]	2004	36	0.6	HG	NA	No	Q+ALND	NA	18 alive	NA	NA	NA
22			55	1.1	IG	Na	No	Q+ALND	NA	3 alive	NA	NA	NA
23			54	1.5	LG	NA	No	Q+ALND	NA	13 alive	NA	NA	NA
24			29	0.8	LG	NA	No	L	NA	90 alive	NA	NA	NA
25			80	0.5	LG	NA	No	L	NA	5 alive	NA	NA	NA
26	Gomez−Aracil *et al*. [25]	2006	69	6	HG	Yes	No	MRM	CT	54 alive	+−	NA	NA
27	Horii *et al*. [26]	2006	54	2.5	LG	No	No	MRM	HT	36 alive	+−	−	NA
28^b^	*Hornychova* *et al.* [27]	2007	30	8	LG	No	No	MRM	RT+CT	60 alive	−−	−	NA
29			63	1.8	HG	No	No	MRM	RT+CT	18 alive	−−	−	NA
30^b^	*Camelo-Piragua* *et al.* [8]	2009	49	> 5, multiple microinvasive foci with extensive in situ	IG	Yes	No	MRM	CT	8 alive	−−	−	+ (del.11q21)
31^b^	*Basbug* *et al.* [28]	2011	69	10	HG	No	No	MRM	RT+CT	12 alive	−−	−	NA
32	Turk *et al*. [29]	2013	40	5.5	NA	Yes	No	MRM	CT	5 alive	−−	−	NA
33^b^	*Palermo* *et al.* [30]	2013	80	4	HG	No	No	NA	NA	NA	−−	NA	NA
34	Fujino *et al*. [31]	2016	71	1.7	IG	No	No	SM	NA	NA	−−	−	− (RT−PCR)
35	Cheng *et al*. [32]	2017	61	3	LG	No	No	SM	No	4 alive	++	−	NA
36			66	1.3	LG	No	No	SM	No	9 alive	+−	−	NA
37			49	1.5	LG	No	No	MRM	No	41 alive	−−	−	NA
38			39	1.5	LG	Yes	No	MRM	No	156 alive	++	−	NA
39	Sherwell-Cabello *et al*. [33]	2017	86	6	LG	No	No	MRM	No	3 alive	−−	−	NA
40^b^	*Burghel* *et al.* [34]^a^	2018	73	< 2	LG	No	No	L	No	50 alive	NA	NA	NA
41	GR Bean *et al*. [6]	2018	49	5	IG	Yes	No	MRM	CT	12 alive	−−	−	+ (FISH, RT−PCR)
42			53	1.6	LG	No	No	L	RT	16 alive	−−	−	+ (FISH, NGS, RT−PCR)
43	Mingfei Yan *et al*. [5]ª	2019	60	1.9	LG	NA	No	L	NA	60 alive	−−	−	+ (FISH)
44	Ru-Pei Ye *et al*. [35]	2020	42	2.6	LG	NA	No	MRM	CT	12 alive	−−	−	NA
45	Fresia Pareja *et al*. [7]^a^	2020	NA	NA	LG	NA	NA	NA	NA	NA	−−	−	+ (FISH, RNA sequencing RT−PCR)
46	Linda Metaxa *et al*. [36]	2020	63	2.1	LG	No	No	L	No	36 alive	+ NA	NA	NA
47	Black *et al*. [37]	2023	65	1.3	LG	No	No	L	RT	30 alive	+−	−	+ (FISH, RT−PCR, NGS)^c^
48	He *et al*. [38]	2023	39	1.2	LG	No	No	NA	NA	24	−−	−	+ (FISH)
49			37	1.2	LG	No	No	NA	NA	30	−−	−	+ (FISH)
50			40	1.5	IG	No	No	NA	NA	12	−−	−	+ (FISH)
51−63^b, d^	*Venetis* *et al.* [39]	2023	41–75 (*n* = 13)	≤ 2/(*n* = 8) 2.1–5/(*n* = 2) > 5/(*n* = 1) NA/(*n* = 3)	LG (*n* = 10) HG (*n* = 3)	No (*n* = 11) Yes (*n* = 1) NA (*n* = 1)	No (*n* = 10) Yes (*n* = 2) NA (*n* = 1)	NA (*n* = 13)	NA (*n* = 13)	NA (*n* = 13)	+(2/13)/−(13/13)	−(13/13)	−(10/10); (FISH) 8/13 (NGS)
64	Present case	2023	58	2	HG	No	No	SM	CT+TT	61 alive	+−	+	− (FISH)

The table summarizes the 64 cases of breast MECs so far reported in literature, with the addition of our case. Reports are listed in chronological order including available information about grading, regional and distant metastasis, therapy, follow-up, receptors status, and molecular analysis. A clear correlation between grading, distant metastasis, and deaths of disease can be observed. Relatively few data are available about molecular analysis, which was performed only in most recent cases. In italics are shown the cases that report the presence of mature squamous cells, intercellular bridges, and/or squamous pearl formation; therefore, not being fully consistent with the current diagnostic criteria of the WHO. The table has been adapted from Ru-Pei Ye *et al*. [35] and Murat Basbug *et al*. [28]

*NA* not available, *RM* radical mastectomy, *MRM* modified radical mastectomy, *SM* simple mastectomy, *Q* quadrantectomy, *L* lumpectomy, *ALND* axillary lymph node dissection, *RT* radiotherapy, *CT* chemotherapy, *HT* hormonal therapy (tamoxifen or aromatase inhibitor), *TT* targeted therapy, *DOD* died of disease, *DOR* died of other reasons, *RT–PCR* reverse transcriptase polymerase chain reaction, *FISH* fluorescence *in situ* hybridization, *NGS* next-generation sequencing (DNA)

^a^Updated information obtained by correspondent authors via email

^b^Cases in which presence of mature squamous cells, intercellular bridges and/or squamous pearls formation is reported

^c^*Case 47 report CRTC3:MAML2 translocation*

^d^Venetis *et al.* report presence of squamous differentiation in 4/13 cases, and absence of true keratinization in all cases of the series (*n* = 13); FISH was performed in 10 of the 13 cases, in 8/13 cases NGS data were also available

Furthermore, the unusual finding of *HER2* amplification prompted us to combine anti-HER2 therapy with backbone a-CT. To the best of our knowledge, no HER2-positive cases of MEC-b or of other salivary gland-like tumors of the breast have been reported in the literature so far, except for one sporadic secretory carcinoma of the breast [40, 41]. On the contrary, about 5% MEC-sg may show HER2 amplification, which may relate to differentiation grade [42, 43]. Therefore, we surmise that our case might be consistent with this observation. Interestingly, about 1/6 to 1/8 of MEC-b belong to the category of the so-called ER low-positive BC, defined by ER expression in < 10% of the tumor cells [44], a feature shared also with other salivary gland-like tumors of the breast [40, 45–47]. The use of endocrine therapy in these cases is highly debated and should be individually discussed [48].

MEC-b is characterized by a mixture of epidermoid, intermediate, and mucinous neoplastic cells. Mucinous differentiation may be inconspicuous, especially in high-grade tumors. Presence of true keratinization and/or squamous pearls formation should prompt to consider another diagnosis (in other words, metaplastic carcinoma with adenosquamous pattern) [4]. To note overt keratinization is accepted in MEC-sg, perhaps explaining why in ~ 10 old MEC-b cases a mature squamous cell component is described (Table 1). As suggested here, the diagnosis of MEC-b remains extremely challenging, especially on diagnostic biopsies. Pathologists should be aware of this rare entity whenever a mixture of intermediate and large eosinophilic cells associated with mucinous differentiation is observed. Immunohistochemistry to confirm the presence of the typical “zoning pattern” is helpful [4–6].

The differential diagnosis is broad and includes apocrine carcinoma, metaplastic adenosquamous carcinoma, mucinous carcinoma, mucinous cystadenocarcinoma, and a metastatic MEC-sg. However, an *in situ* component should exclude the latter [4]. We excluded also the possibility of a late recurrence of the primary BC because of the lack of MEC elements, the strong hormone receptor expression in 1996, and the presence of an *in situ* component with MEC features in the current tumor, supporting the diagnosis of a second primary.

To date molecular analysis has been reported in 21 MEC-b, of which seven harbored *CRTC1-MAML2* and one harbored *CRTC3-MAML2* translocation (Table 1) [5–7, 37–39]. Remarkably, the majority of positive cases were either low or intermediate grade. Likewise in MEC-sg, *MAML2* translocation seems to be the most frequent recurrent genetic alteration also in MEC-b (*n* = 9/21, 43% prevalence). However, we were not able to detect *MAML2* translocation by FISH, which did not prevent us to confirm the diagnosis because of clear-cut morphology. Similarly Venet *et al*. did not detect *MAML2* rearrangements in any of the 10 MEC-b tested by FISH, questioning the diagnostic value of this molecular hallmark in MEC-b. Notably, three low-grade MEC-b were not tested in their series [39]. Techniques like RT–PCR and FISH taken individually may have low sensitivity due to technical issues (for example, polymerase errors, small deletions, and so on) as compared with more sensitive techniques like RNA sequencing. Conversely, when considering our case, we may speculate a causal correlation with poor differentiation grade as suggested in MEC-sg [49].

## Conclusions

MEC-b is a very rare entity. Diagnosis on small diagnostic biopsies may be challenging. Strict application of WHO criteria is desirable, as well as standard evaluation of receptor status for best patient care.

## Data Availability

All data generated or analyzed during this study are included in this published article.

## Abbreviations

a-CT: Adjuvant chemotherapy
AR: Androgen receptor
BC: Breast cancer
DCIS: Ductal carcinoma *in situ*
ER: Estrogen receptor
HER2: Human epidermal growth factor receptor 2
IBC-NST: Invasive breast carcinoma of no special type
IHC: Immunohistochemistry
MAML2: Mastermind-like transcriptional coactivator 2
MEC-b: Mucoepidermoid carcinoma of the breast
MEC-sg: Mucoepidermoid carcinoma of the salivary glands
PR: Progesterone receptor
TNBC: Triple-negative breast carcinoma
WHO: World health organization
